# Organs-at-risk dose constraints in head and neck intensity-modulated radiation therapy using a dataset from a multi-institutional clinical trial (JCOG1015A1)

**DOI:** 10.1186/s13014-022-02105-3

**Published:** 2022-07-28

**Authors:** Masahiro Inada, Yasumasa Nishimura, Satoshi Ishikura, Kazuki Ishikawa, Naoya Murakami, Takeshi Kodaira, Yoshinori Ito, Kazuhiko Tsuchiya, Yuji Murakami, Junichi Saito, Tetsuo Akimoto, Kensei Nakata, Michio Yoshimura, Teruki Teshima, Takashi Toshiyasu, Yosuke Ota, Toshiyuki Minemura, Hidetoshi Shimizu, Masahiro Hiraoka

**Affiliations:** 1 Department of Radiation Oncology, Kindai University Faculty of Medicine, 377-2 Onohigashi, Osakasayama, Osaka 589-8511 Japan; 2 Division of Radiation Oncology, Tokyo Bay Makuhari Clinic for Advanced Imaging, Cancer Screening, and High-Precision Radiotherapy, Chiba, Japan; 3 Department of Radiation Oncology, Nara Prefecture General Medical Center, Nara, Japan; 4 Department of Radiation Oncology, National Cancer Center Hospital, Tokyo, Japan; 5 Department of Radiation Oncology, Aichi Cancer Center Hospital, Aichi, Japan; 6 Department of Radiation Oncology, Showa University School of Medicine, Tokyo, Japan; 7 Department of Radiation Oncology, Otaru General Hospital, Hokkaido, Japan; 8 Department of Radiation Oncology, Hiroshima University Hospital, Hiroshima, Japan; 9 Division of Radiation Oncology, Department of Radiology, Faculty of Medicine, University of Toyama, Toyama, Japan; 10 Department of Radiation Oncology, Sapporo City General Hospital, Hokkaido, Japan; 11 Department of Radiation Oncology and Image-Applied Therapy, Kyoto University, Kyoto, Japan; 12 Osaka Heavy Ion Therapy Center, Osaka, Japan; 13 Department of Radiation Oncology, Cancer Institute Hospital of Japanese Foundation for Cancer Research, Tokyo, Japan; 14 Department of Radiation Oncology, Hyogo Cancer Center, Hyogo, Japan; 15 Institute for Cancer Control, National Cancer Center Hospital, Tokyo, Japan; 16 Department of Radiation Oncology, Japanese Red Cross Wakayama Medical Center, Wakayama, Japan; 17 Department of Radiation Oncology, National Cancer Center Hospital East, Chiba, Japan

**Keywords:** Head and neck carcinoma, Intensity-modulated radiation therapy, Chemoradiation therapy, Dose constraints, Prospective trial

## Abstract

**Background:**

JCOG1015A1 is an ancillary research study to determine the organ-specific dose constraints in head and neck carcinoma treated with intensity-modulated radiation therapy (IMRT) using data from JCOG1015.

**Methods:**

Individual patient data and dose-volume histograms of organs at risk (OAR) were collected from 74 patients with nasopharyngeal carcinoma treated with IMRT who enrolled in JCOG1015. The incidence of late toxicities was evaluated using the cumulative incidence method or prevalence proportion. ROC analysis was used to estimate the optimal DVH cut-off value that predicted toxicities.

**Results:**

The 5-year cumulative incidences of Grade (G) 1 myelitis, ≥ G1 central nervous system (CNS) necrosis, G2 optic nerve disorder, ≥ G2 dysphagia, ≥ G2 laryngeal edema, ≥ G2 hearing impaired, ≥ G2 middle ear inflammation, and ≥ G1 hypothyroidism were 10%, 5%, 2%, 11%, 5%, 26%, 34%, and 34%, respectively. Significant associations between DVH parameters and incidences of toxicities were observed in the brainstem for myelitis (D1cc ≥ 55.8 Gy), in the brain for CNS necrosis (D1cc ≥ 72.1 Gy), in the eyeball for optic nerve disorder (Dmax ≥ 36.6 Gy), and in the ipsilateral inner ear for hearing impaired (Dmean ≥ 44 Gy). The optic nerve, pharyngeal constrictor muscle (PCM), and thyroid showed tendencies between DVH parameters and toxicity incidence. The prevalence proportion of G2 xerostomia at 2 years was 17 versus 6% (contralateral parotid gland Dmean ≥ 25.8 Gy vs less).

**Conclusions:**

The dose constraint criteria were appropriate for most OAR in this study, although more strict dose constraints might be necessary for the inner ear, PCM, and brainstem.

**Supplementary Information:**

The online version contains supplementary material available at 10.1186/s13014-022-02105-3.

## Background

Intensity-modulated radiation therapy (IMRT), an advanced technique in external beam radiation therapy, has been widely used for head and neck carcinoma [1]. IMRT can deliver a more conformal dose to targets with reduced exposure to normal organs, which has improved xerostomia resulting from head and neck radiation therapy compared with 3D-conformal radiation therapy (3D-CRT) [2–6].

The Quantitative Analyses of Normal Tissue Effects in the Clinic (QUANTEC) study, which reviewed and summarized normal tissue toxicity data from clinical trials, provided basic data of dose-volume constraints and clinical goals during radiation treatment planning for physicians and radiation oncologists [7, 8]. While much of the evidence forming this literature was based on clinical trials or clinical practice using 3D-CRT, these dose-volume constraints data should be revised in the era of IMRT.

The Japan Clinical Oncology Group (JCOG) study, a single-arm phase II trial, was conducted to investigate adaptive two-step IMRT for nasopharyngeal carcinoma (JCOG1015, UMIN-CTR: UMIN000005448) with more than 3 years’ follow-up and showed a 3-year overall survival rate of 88% [9]. In the present study, the association between dose-volume histogram (DVH) parameters of normal tissues and late toxicities reported from patients enrolled in JCOG1015 were analyzed. The aim of this study was to review the dose-volume constraints used in JCOG1015. IMRT specific dose-volume constraints for the area of head and neck radiation therapy were investigated.

## Methods

The protocol of this ancillary study (JCOG1015A1) was approved by the JCOG Protocol Review Committee and the institutional review board of Kindai University and National Cancer Center. All patients provided written informed consent for including to JCOG1015 and for secondary use of data. Between 2011 and 2014, 75 patients were enrolled in JCOG1015. One patient who refused chemoradiotherapy was excluded from this analysis, and overall, the remaining 74 patients were included in this study. Patients’ characteristics are shown in Table 1.

**Table 1:** Patient and treatment characteristics (n = 74)

Gender	Female/male	15/59
Age	Median	55
	Range	28–75
PS	0/1	56/18
WHO type	I/II/III	7/33/34
Clinical stage	II/III/IVA/IVB	16/33/13/12
T stage	1/2/3/4	22/15/22/15
N stage	0/1/2/3	5/26/31/12
Courses of concurrent CT	2/3	17/57
Courses of adjuvant CT	0/1/2/3	15/5/15/39

*CT* Chemotherapy

All 74 patients were treated with adaptive two-step IMRT at a total dose of 70 Gy and at least two courses of concurrent chemotherapy (cisplatin 80 mg/m^2^ over 3 weeks). For all patients, CT planning was performed twice before the initial whole-neck plan (plan-1) of 46 Gy/23 fractions, and at the fourth week for the boost plan (plan-2) of 24 Gy/12 fractions to the high-risk clinical target volume. Details of treatment procedures and outcomes were previously reported [9]. At the time of the analysis of JCOG1015A1, 62 of 74 patients were alive with at least 3 years’ follow-up, and the median follow-up period was 50 months.

In JCOG1015, all IMRT plans were centrally reviewed by two radiation oncologists, and DVH parameters of organs at risk (OAR) were collected. Our goals on the DVH used in JCOG1015 are shown in Table 2.The doses for the parotid gland and inner ear were recorded individually for the left and right sides. In addition, the dose constraints for the parotid gland and inner ear should be achieved on at least one side. No dose constraint was established for the thyroid gland. A planning organ-at-risk volume (PRV) margin of 3–5 mm was added to the spinal cord. PRV margins of at least 1–2 mm were added to the brain, brainstem, optic nerves, and inner ears, although no PRV margins were added to the parotid glands and thyroid gland. Cranial and caudal boundaries of the middle and lower pharyngeal constrictor muscle were the cranial edge of the third cervical vertebrae and the caudal edge of the cricoid cartilage. The dose limitations of the spinal cord PRV and PTV were achieved with the highest priority. In both plan-1 and plan-2, the optimizations were performed as a 70-Gy plan, and the dose constraints were met in each 70-Gy plan. The total DVH parameters of each OAR, such as Dmax, D1cc, and Dmean, were calculated as follows:

$${\text{D}}\left( {{\text{total}}} \right) \, = {\text{ D}}\;\left( {{\text{plan}} - {1}} \right) \, \times { 46}/{7}0 \, + {\text{ D}}\left( {{\text{plan}} - {2}} \right) \, \times { 24}/{7}0$$

**Table 2:** Dose constraints for OAR in the JCOG1015 protocol

OARs	Parameter	Goal	Acceptable
Spinal cord PRV	Dmax	< 50 Gy	< 54 Gy
	D1cc	< 46 Gy	< 50 Gy
Brain PRV	Dmax	< 70 Gy	< 74 Gy
	D1cc	Not stated	< 70 Gy
Brainstem PRV	Dmax	< 54 Gy	< 64 Gy
	D1cc	Not stated	< 60 Gy
Optic nerve PRV	Dmax	< 50 Gy	< 54 Gy
Eyeball	Dmax	< 40 Gy	< 45 Gy
Lens	Dmean	< 6 Gy	< 10 Gy
Parotid glands (at least 1 gland)	Dmedian	< 20 Gy	< 24 Gy
	Dmean	< 26 Gy	< 30 Gy
Middle and lower pharyngeal constrictor muscle	Dmean	< 54 Gy	< 60 Gy
Larynx	Dmean	< 45 Gy	< 50 Gy
Inner ears PRV (at least 1 ear)	Dmean	< 45 Gy	< 50 Gy
Thyroid	Dmean	Not stated	Not stated

*OAR* Organs at risk, *PRV* planning organ-at-risk volume

Late toxicities were evaluated every 6 months and graded according to the Common Terminology Criteria for Adverse Events (CTCAE) version 4.0 [10]. We evaluated noteworthy late toxicities as follows: ≥ G2 hearing impaired, ≥ G2 middle ear inflammation, ≥ G1 hypothyroidism, ≥ G1 cataract, ≥ G1 optic nerve disorder, ≥ G2 dysphagia, ≥ G2 laryngeal edema, ≥ G1 myelitis, ≥ G1 central nervous system (CNS) necrosis, and ≥ G2 xerostomia. The incidence of late toxicities, except for xerostomia, were estimated using the cumulative incidence function and any cause of death was defined as a competing risk. The prevalence of xerostomia was defined as the proportion of patients with ≥ G2 xerostomia for all surviving patients at 1, 2, and 3 years. Time-to-event type endpoints were calculated from the date of the initiation of IMRT.

### Statistical analyses

To investigate the dose-volume effect association of IMRT, patients were divided into two groups (high- and low-dose groups) according to the optimal cut-off values determined in this study. The cut-off values in each OAR were estimated based on Youden’s index. The area under the curve (AUC) was calculated by receiver operating characteristic (ROC) analysis (results of ROC analysis were shown in Additional file 1: Table S1). Cumulative incidence functions of late toxicities (except for xerostomia) were compared between the high- and low-dose groups using Gray’s test. The prevalence proportion of xerostomia was compared between groups using Pearson’s chi-squared test. A p-value of < 0.05 was considered significant. All analyses were performed with EZR v1.53 (Saitama Medical Center, Jichi Medical University, Saitama, Japan), which is a graphical user interface for R v4.0.2 (The R Foundation for Statistical Computing, Vienna, Austria) and BellCurve for Excel v3.20 (Social Survey Research Information Co., Ltd, Tokyo, Japan).

## Results

The incidence of late toxicities, DVHs, and estimated cut-off values are shown in Table 3.

**Table 3:** Comparison of dose metrics by late toxicities and cut-off value

End point	Number of event	5-year cumulative incidence (%)	Normal tissue	Dose metric	Without toxicities (Gy)	With toxicities (Gy)	Estimated cut-off value (Gy)
					Median (range)	Median (range)	
Myelitis G1	7	10	Spinal cord	Dmax	49.1 (39.4–53.5)	48.4 (44.3–56.7)	45.5
				D1cc	43.1 (31.2–48.2)	41.4 (39.7–49.6)	49.1
Myelitis G1	7	10	Brainstem	Dmax	55.9 (46.5–68.3)	59.7 (47.2–62.3)	59.7
				D1cc	49.7 (36.1–60.6)	55.9 (41.6–58.8)	55.8
CNS necrosis ≥ G1	2	5	Brain	Dmax	73.2 (64.9–81.5)	75.3 (74.6–76.1)	74.5
				D1cc	67.6 (47.5–76.3)	72.7 (72.1–73.3)	72.1
Optic nerve disorder G2	1	2	Optic nerve	Dmax	43.2 (5.1–78.9)	53.4	53.3
Optic nerve disorder G2	1	2	Eye ball	Dmax	26.7 (3.5–50.6)	36.6	36.6
Cataract G1	2	3	Lens	Dmean	4.6 (1.8–20.1)	3.6 (1.8–5.3)	1.8
Dysphagia ≥ G2	9	11	PCM	Dmean	45.9 (27.2–62.9)	44.6 (33.0–56.4)	41.2
Laryngeal edema ≥ G2	2	5	Larynx	Dmean	39.3 (24.4–66.8)	43.6 (37.4–49.9)	49.0
Hearing impaired ≥ G2	17	26	Inner ear (contralateral)	Dmean	37.2 (23.9–40.7)	39.4 (26.5–42.5)	37.6
Hearing impaired ≥ G2	17	26	Inner ear (ipsilateral)	Dmean	40.6 (24.3–65.9)	44.2 (31.3–72.0)	44.0
Middle ear inflammation ≥ G2	25	34	Inner ear (ipsilateral)	Dmean	41.2 (27.7–69.3)	42.6 (24.3–72.0)	51.5
Hypothyroidism ≥ G1	24	34	Thyroid	Dmean	46.7 (2.9–65.5)	47.1 (10.3–69.7)	45.6
Hypothyroidism G2	17	27		Dmean	47.3 (2.9–69.7)	46.9 (10.4–61.5)	45.6

*PCM* Pharyngeal constrictor muscle,* CNS* central nervous system

**Fig. 1:**
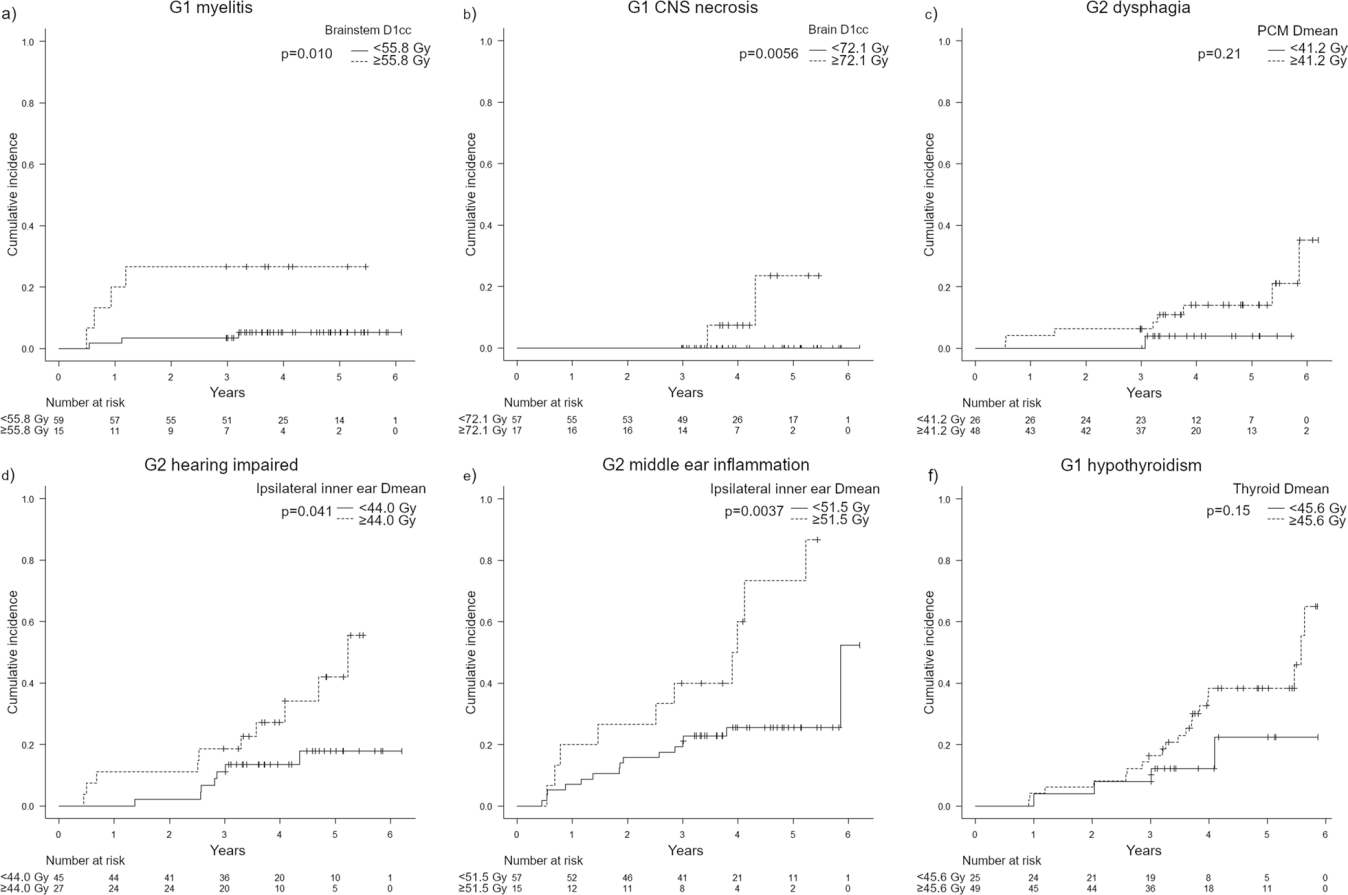
Cumulative incidence curves of late toxicities for G1 myelitis by brainstem D1cc (**a**), G1 CNS necrosis by brain D1cc (**b**), G2 dysphagia by PCM Dmean (**c**), G2 hearing impaired by ipsilateral inner ear Dmean (**d**), G2 middle ear inflammation by ipsilateral inner ear Dmean (**e**), and G1 hypothyroidism by thyroid Dmean (**f**)

The cut-off values were estimated in the brainstem, brain, optic nerve, eyeball, pharyngeal constrictor muscle (PCM), inner ear, and thyroid (≥ G1 hypothyroidism). In the spinal cord, lens, larynx, and thyroid (G2 hypothyroidism), two cumulative incidence curves divided by the cut-off value almost overlapped or the incidence proportion was higher in the low-dose group. The 5-year cumulative incidence of toxicity for the entire patient group is shown in Table 3, and that for each dose group divided by the cut-off values is shown in Table 4. There were statistically significant differences between the two dose groups regarding G1 myelitis, ≥ G1 CNS necrosis, G2 optic nerve disorder, ≥ G2 hearing impaired, and ≥ G2 middle ear inflammation. However, there was only one case of G2 optic nerve disorder. The cumulative incidence of ≥ G2 dysphagia in the PCM Dmean ≥ 41.2 Gy group and of ≥ G1 hypothyroidism in the thyroid Dmean ≥ 45.6 Gy group tended to be higher than those in the low-dose groups. The cumulative incidence curves of G1 myelitis, ≥ G1 CNS necrosis, ≥ G2 dysphagia, ≥ G2 hearing impaired, ≥ G2 middle ear inflammation, and ≥ G1 hypothyroidism in each dose group are shown in Fig. 1.

**Table 4:** The 3- and 5-year cumulative incidence rate of late toxicities by dose metrics

End point	Normal tissue	Dose metric	Number	3 year (95% CI)	5 year (95% CI)	*p*-value
Myelitis G1	Brainstem	D1cc ≥ 55.8 Gy	15	27% (8–50)	27% (8–50)	0.010
		D1cc < 55.8 Gy	59	3% (0–10)	5% (1–13)	
CNS necrosis ≥ G1	Brain	D1cc ≥ 72.1 Gy	17	0% (N/A)	24% (3–56)	0.0056
		D1cc < 72.1 Gy	57	0% (N/A)	0% (N/A)	
Optic nerve disorder G2	Optic nerve	Dmax ≥ 53.3 Gy	13	0% (N/A)	8% (0–32)	0.051
		Dmax < 53.3 Gy	61	0% (N/A)	0% (N/A)	
Optic nerve disorder G2	Eye ball	Dmax ≥ 36.6 Gy	9	0% (N/A)	13% (0–43)	0.012
		Dmax < 36.6 Gy	65	0% (N/A)	0% (N/A)	
Dysphagia ≥ G2	PCM	Dmean ≥ 41.2 Gy	48	6% (2–16)	14% (6–26)	0.21
		Dmean < 41.2 Gy	26	0% (N/A)	4% (0–17)	
Hearing impaired ≥ G2	Inner ear (contralateral)	Dmean ≥ 37.6 Gy	39	21% (10–34)	35% (17–53)	0.062
		Dmean < 37.6 Gy	35	9% (2–21)	17% (6–33)	
Hearing impaired ≥ G2	Inner ear (ipsilateral)	Dmean ≥ 44.0 Gy	27	19% (7–40)	42% (20–63)	0.041
		Dmean < 44.0 Gy	45	11% (4–22)	18% (7–32)	
Middle ear inflammation ≥ G2	Inner ear (ipsilateral)	Dmean ≥ 51.5 Gy	18	50% (22–73)	73% (34–92)	0.0037
		Dmean < 51.5 Gy	54	19% (10–30)	25% (15–38)	
Hypothyroidism ≥ G1	Thyroid	Dmean ≥ 45.6 Gy	49	16% (8–30)	38% (24–53)	0.15
		Dmean < 45.6 Gy	21	8% (1–23)	22% (6–46)	

CNS, central nervous system; CI, confidence interval

The proportion of patients with G2 xerostomia by parotid gland dose are shown in Table 5, and were 31%, 17% and 13% at 1, 2, and 3 years, respectively, in the contralateral parotid gland Dmean ≥ 25.8 Gy group, and 20%, 6%, and 6%, respectively, in the < 25.8 Gy group. A reverse association was observed between the ipsilateral parotid gland Dmedian and G2 xerostomia.

**Table 5:** Proportion of patients with G2 xerostomia at 1, 2, and 3 years by parotid gland dose

	Dose (Gy)	At 1 year		At 2 years		At 3 years	
		Proportion	*p* value	Proportion	*p* value	Proportion	*p* value
Dmedian (contralateral)	≥ 19.2 (n = 37) < 19.2 (n = 36)	34% (n = 12) 18% (n = 6)	0.11	17% (n = 6) 6% (n = 2)	0.16	12% (n = 4) 7% (n = 2)	0.49
Dmean (contralateral)	≥ 25.8 (n = 38) < 25.7 (n = 36)	31% (n = 11) 20% (n = 7)	0.27	17% (n = 6) 6% (n = 2)	0.14	13% (n = 4) 6% (n = 2)	0.37
Dmedian (ipsilateral)	≥ 25.3 (n = 33) < 25.3 (n = 39)	19% (n = 6) 30% (n = 11)	0.33	3% (n = 1) 17% (n = 6)	0.073	0% (n = 0) 15% (n = 5)	0.026
Dmean (ipsilateral)	≥ 36.9 (n = 24) < 36.9 (n = 46)	22% (n = 5) 26% (n = 12)	0.69	4% (n = 1) 13% (n = 6)	0.25	0% (n = 0) 12% (n = 5)	0.092
Dmedian (bilateral)	≥ 22.0 (n = 40) < 22.0 (n = 32)	26% (n = 10) 23% (n = 7)	0.78	11% (n = 4) 10% (n = 3)	0.98	5% (n = 2) 12% (n = 3)	0.38
Dmean (bilateral)	≥ 29.8 (n = 36) < 29.8 (n = 37)	24% (n = 8) 25% (n = 9)	0.94	10% (n = 3) 13% (n = 4)	0.75	3% (n = 1) 13% (n = 4)	0.21

## Discussion

In this study, the association between late toxicities and DVH parameters for patients with advanced nasopharyngeal cancer treated by IMRT with chemotherapy were analyzed using clinical trial (JCOG1015) data of 74 patients with advanced nasopharyngeal cancer. In this analysis, a significant association between the incidence of late toxicities and DVH parameters were observed in the brainstem, brain, and inner ear. In addition, trends towards a higher incidence of toxicities in the high-dose group were observed in the PCM and thyroid (G1 hypothyroidism). These results should be investigated in future clinical trials and in clinical practice for head and neck IMRT.

Ototoxicity is most common complication after radiation therapy for nasopharyngeal cancer. Hearing impaired was a frequent late toxicity in JCOG1015. Because the inner ear structures are close to the clivus and retropharyngeal lymph area, irradiation doses to the inner ear tend to be higher than those for other head and neck carcinomas. In addition, combination of this treatment with high-dose cisplatin also carries risk of ototoxicity. In the present study, there was no significant association between the incidence of ≥ G2 hearing impaired and the course of cisplatin (data not shown). Impaired hearing after head and neck chemoradiation therapy is well documented, and dose–response relationships were reported in several papers [11–14]. In the present analysis, ≥ G2 hearing impaired was significantly associated with the mean dose to the ipsilateral inner ear ≥ 44.0 Gy (p = 0.041). This result was similar to Lee’s guidelines and QUANTEC [8, 15]. Middle ear inflammation (otitis media) was more frequently observed in JCOG1015. The mechanism of otitis media with effusion (OME) after radiation therapy was hypothesized to be direct radiation damage to middle ear and/or nasopharyngitis, rhinitis, and sinusitis [16]. The radiation dose to the middle ear cavity was reported to be associated with the risk of OME [17, 18]. In JCOG1015, the middle ear dose was not evaluated; therefore, the inner ear dose was analyzed as OAR for middle ear inflammation instead. There was a significant association between the incidence of middle ear inflammation and the inner ear dose. The incidence of middle ear inflammation was relatively high in this study; thus, middle ear dose reduction seems to be important [16, 17].

CNS disorders are the most important complications and should be carefully monitored in head and neck radiation therapy. In JCOG1015, G1 myelitis and ≥ G1 CNS necrosis were observed in seven (9%) and two (3%) patients, respectively. G1 myelitis, so-called “Lhermitte’s sign”, is reversible demyelination of the cervical or thoracic spine after radiation therapy. The incidence of Lhermitte’s sign after IMRT has been reported to be between 3.6% and 13% [19]. The incidence of myelitis did not depend on spinal cord dose but brainstem dose (Table 4). These results may be attributed as the highest priority of the spinal cord PRV. No previous studies have described the association between brainstem dose and Lhermitte’s sign. Radiation-induced brainstem injury is mainly noted as brainstem necrosis. Severe brainstem necrosis may cause cranial nerve and cerebellar injury symptoms, and the dose constraint of the brainstem was recommended to be under 54 Gy at the maximum dose [7, 15, 20], which was similar to the result of the present study. In JCOG1015, no severe symptoms caused by CNS necrosis were observed, although the incidence of Lhermitte’ sign may be caused by mild brainstem injury.

Dysphagia is the most important toxicity after radiation therapy and is associated with poor quality of life. Long-term follow-up of RTOG 91–11 revealed that concurrent chemoradiation therapy improved locoregional control but not survival compared with radiation therapy alone or induction chemotherapy [21]. This might be caused by swallowing dysfunction and aspiration. Swallowing organ-sparing IMRT is promising to resolve this issue [22]. In JCOG1015, the middle and inferior PCM dose was evaluated. Patients who received ≥ 41.2 Gy (mean) to the PCM tended to have ≥ G2 dysphagia, and this value was lower than the protocol goal (Table 2). In a previous report that described the association between PCM dose and dysphagia, PCM dosimetry and the incidence of dysphagia was relatively high compared with JCOG1015 [23–25]. In the QUANTEC database, the Dmean of the whole pharyngeal constrictor was recommended to be lower than 50 Gy. There are no data evaluating the association between the middle and lower PCM and dysphagia in IMRT for nasopharyngeal carcinoma. It seems to be appropriate to reduce the mean middle and lower PCM dose to less than 41.2 Gy.

Hypothyroidism after radiation therapy to the head and neck has been reported to occur in 10% to 50% of patients [26]. Previous reports have described that hypothyroidism is associated with doses of 40–50 Gy [26–31]. In the current study, only Dmean was evaluated. There was no significant dose–response relationship in hypothyroidism. Most cases of hypothyroidism occurred at 3 years after radiation therapy, especially in the high-dose group (Fig. 1), and thus longer follow-up is needed to evaluate the dose–response relationship of hypothyroidism.

Parotid gland dose reduction is the most beneficial change from conventional radiation therapy to IMRT [5, 32–35]. The incidence of ≥ G2 xerostomia at 2–5 years ranged from 0 to 22% in previous studies [5, 34, 35]. In JCOG1015, 9% of patients developed G2 xerostomia at 3 years, which seemed to be acceptable. The proportion of G2 xerostomia at 2 years after radiation therapy seemed to be higher in the high-dose group (contralateral parotid gland Dmean ≥ 25.8 Gy versus < 25.8 Gy: 17 versus 6%), but the difference was not significant (*p* = 0.14). In contrast, it was unclear why a significant inverse dose–response relationship was observed between the ipsilateral parotid gland dose and xerostomia.

Overall, the dose constraints used in JCOG1015, which was based on the QUANTEC criteria, generally seemed to be appropriate. In terms of the high incidence of ototoxicity and dose–response relationships, more careful dose reductions are required for auditory organs. Evaluation of middle ear DVH parameters are also important. Dose constraint of the brainstem in JCOG1015 (Dmax < 54 Gy) seemed to be safe, although this constraint could not be met frequently. The higher priority for the brainstem dose limitation was needed to reduce the Lhermitte sign. In terms of dysphagia, the cut-off value for the PCM dose was estimated (Dmean < 41.2 Gy) to be lower than that of the protocol goal (Dmean < 54 Gy). In the future, increased dose reduction for the PCM seems to be desirable.

There are several limitations of this study. First, it was difficult to show a significant dose–response relationship because of the small number of patients and the relatively short-term follow-up. Especially, late-phase incidences were noted in dysphagia and hypothyroidism. In contrast, the small number of late toxicities also limited the statistical power in this study. There were only one and two cases of optic nerve disorder and CNS necrosis, respectively. Although statistically significant cut-off values could be found for these organs, it was not enough to determine the clinically useful dose constraints. Second, toxicity evaluations were undertaken using CTCAE. It is sometimes inevitable that the use of these criteria includes subjective features, especially in dysphagia, xerostomia, and hearing loss. Objective clinical examinations will provide more persuasive data in the future [11, 23, 33, 34]. However, toxicity information was screened prospectively every 6 months in JCOG1015. Thus, we believe that the reliability of the toxicity grade is maintained. Third, all patients were treated by the adaptive two-step IMRT method in this study. The sequential two-step method has two different CT contour sets; thus, the dose parameters of OAR were possibly inaccurate. However, the advantage of the adaptive method is its ability to adjust to an anatomical and tumor responsive change during IMRT [36]. We believe that this inaccuracy could be negligible.

## Conclusions

In conclusion, the dose constraint criteria used in JCOG1015 seems to be appropriate for most OAR. To reduce late toxicity, the dose constraints of the PCM should be decreased. Furthermore, brainstem and inner ear dose constraints should be undertaken with higher priority.

## Supplementary Information

**Additional file 1**. Table S1. Results of receiver operator characteristic analysis in each late toxicity.

## Data Availability

Not applicable.

## Abbreviations

JCOG: Japanese clinical oncology group
IMRT: Intensity-modulated radiation therapy
DVH: Dose-volume histogram
OAR: Organs-at-risk
ROC: Receiver operating characteristic
CNS: Central nerve system
PCM: Pharyngeal constrictor muscle
3D-CRT: 3-Dimensional conformal radiation therapy
QUANTEC: Quantitative analyses of normal tissue effects in the clinic
CT: Computed tomography
PRV: Planning organ-at-risk volume
PTV: Planning target volume
CTCAE: Common terminology criteria for adverse events
OME: Otitis media with effusion
