# Supplementary material for: Organs-at-risk dose constraints in head and neck intensity-modulated radiation therapy using a dataset from a multi-institutional clinical trial (JCOG1015A1)
Source: Radiat Oncol. 2022 Jul 28;17:133. doi: 10.1186/s13014-022-02105-3 (PMC9331577; doi:10.1186/s13014-022-02105-3)
Supplement: Supplementary file 1 — Additional file 1. Table S1. Results of receiver operator characteristic analysis in each late toxicity. [file 13014_2022_2105_MOESM1_ESM.docx]

Table S1. Results of receiver operator characteristic analysis in each late toxicity.

| End point | Normal tissue | Dose metric | Cut-off value | Sensitivity | Specificity | AUC |
| --- | --- | --- | --- | --- | --- | --- |
| Myelitis G1 | Spinal cord | Dmax | 45.6 | 0.428 | 0.881 | 0.55 |
|  |  | D1cc | 41.4 | 0.571 | 0.746 | 0.55 |
| Myelitis G1 | Brainstem | Dmax | 59.7 | 0.571 | 0.687 | 0.54 |
|  |  | D1cc | 55.8 | 0.571 | 0.836 | 0.62 |
| CNS necrosis ≥G1 | Brain | Dmax | 74.5 | 1.000 | 0.709 | 0.76 |
|  |  | D1cc | 72.1 | 1.000 | 0.792 | 0.82 |
| Vision impaired G2 | Optic nerve | Dmax | 53.3 | 1.000 | 0.836 | 0.84 |
| Vision impaired G1 | Eye ball | Dmax | 36.6 | 1.000 | 0.890 | 0.89 |
| Cataract G1 | Lens | Dmean | 1.9 | 0.500 | 0.986 | 0.66 |
| Dysphagia ≥G2 | PCM | Dmean | 41.2 | 0.889 | 0.385 | 0.52 |
| Laryngeal edema ≥G2 | Larynx | Dmean | 49.9 | 0.500 | 0.931 | 0.67 |
| Hearing impaired ≥G2 | Inner ear (contralateral) | Dmean | 37.6 | 0.706 | 0.526 | 0.60 |
| Hearing impaired ≥G2 | Inner ear (ipsilateral) | Dmean | 44.0 | 0.588 | 0.691 | 0.64 |
| Middle ear inflammation ≥G2 | Inner ear (ipsilateral) | Dmean | 51.5 | 0.400 | 0.894 | 0.57 |
| Hypothyroidism ≥G1 | Thyroid | Dmean | 45.6 | 0.833 | 0.440 | 0.56 |
| Hypothyroidism G2 | Thyroid | Dmean | 45.6 | 0.823 | 0.403 | 0.52 |
